# Prevalence of pathogenic *BRCA1/2* germline mutations among 802 women with unilateral triple-negative breast cancer without family cancer history

**DOI:** 10.1186/s12885-018-4029-y

**Published:** 2018-03-07

**Authors:** Christoph Engel, Kerstin Rhiem, Eric Hahnen, Sibylle Loibl, Karsten E. Weber, Sabine Seiler, Silke Zachariae, Jan Hauke, Barbara Wappenschmidt, Anke Waha, Britta Blümcke, Marion Kiechle, Alfons Meindl, Dieter Niederacher, Claus R. Bartram, Dorothee Speiser, Brigitte Schlegelberger, Norbert Arnold, Peter Wieacker, Elena Leinert, Andrea Gehrig, Susanne Briest, Karin Kast, Olaf Riess, Günter Emons, Bernhard H. F. Weber, Jutta Engel, Rita K. Schmutzler, on behalf of the German Consortium for Hereditary Breast and Ovarian Cancer (GC-HBOC)

**Affiliations:** 1 Institute for Medical Informatics, Statistics and Epidemiology, University of Leipzig, Leipzig, Germany; 2 Center for Hereditary Breast and Ovarian Cancer and Center for Integrated Oncology (CIO), Medical Faculty, University Hospital Cologne, Kerpener Strasse 34, 50931 Cologne, Germany; 3 German Breast Group, Neu-Isenburg, Germany; 4 Centre for Haematology and Oncology Bethanien, Frankfurt, Germany; 5 Department of Gynecology and Center for Hereditary Breast and Ovarian Cancer, Klinikum rechts der Isar, Technical University Munich (TUM), Munich, Germany; 6 Department of Gynecology and Obstetrics, University Hospital of the Heinrich-Heine University, Düsseldorf, Germany; 7 Institute of Human Genetics, University Hospital, University of Heidelberg, Heidelberg, Germany; 8 Zentrum für Familiären Brust- und Eierstockkrebs, Klinik für Gynäkologie mit Brustzentrum, Charité – Universitätsmedizin Berlin, Berlin, Germany; 9 Department of Human Genetics, Hannover Medical School, Hannover, Germany; 10 Institute of Clinical Molecular Biology/Department of Gynecology and Obstetrics, University Hospital of Schleswig-Holstein, Campus Kiel, Christian-Albrechts University Kiel, Kiel, Germany; 11 Institute of Human Genetics, University Hospital Münster, Münster, Germany; 12 Department of Gynecology and Obstetrics, University Hospital Ulm, Ulm, Germany; 13 Institute of Human Genetics, University Würzburg, Würzburg, Germany; 14 Center for Hereditary Breast and Ovarian Cancer, University Hospital Leipzig, Leipzig, Germany; 15 Department of Gynecology and Obstetrics, Medical Faculty and University Hospital Carl Gustav Carus, Technische Universität Dresden, Dresden, Germany; 16 National Center for Tumor Diseases (NCT), Partner Site Dresden, Dresden, Germany; 17 German Cancer Consortium (DKTK), Dresden, and German Cancer Research Center (DKFZ), Heidelberg, Germany; 18 Institute of Medical Genetics and Applied Genomics, University of Tübingen, Tübingen, Germany; 19 Klinik für Gynäkologie und Geburtshilfe, Universitätsmedizin, Göttingen, Germany; 20 Institute of Human Genetics, University of Regensburg, Regensburg, Germany; 21 Munich Cancer Registry (MCR) of the Munich Tumour Centre (TZM), Institute for Medical Information Processing, Biometry and Epidemiology (IBE), University Hospital of Munich, Ludwig-Maximilians-University (LMU), Munich, Germany

**Keywords:** Hereditary breast and ovarian cancer, *BRCA1*, *BRCA2*, Triple-negative breast cancer

## Abstract

**Background:**

There is no international consensus up to which age women with a diagnosis of triple-negative breast cancer (TNBC) and no family history of breast or ovarian cancer should be offered genetic testing for germline *BRCA1* and *BRCA2* (gBRCA) mutations. Here, we explored the association of age at TNBC diagnosis with the prevalence of pathogenic gBRCA mutations in this patient group.

**Methods:**

The study comprised 802 women (median age 40 years, range 19–76) with oestrogen receptor, progesterone receptor, and human epidermal growth factor receptor type 2 negative breast cancers, who had no relatives with breast or ovarian cancer. All women were tested for pathogenic gBRCA mutations. Logistic regression analysis was used to explore the association between age at TNBC diagnosis and the presence of a pathogenic gBRCA mutation.

**Results:**

A total of 127 women with TNBC (15.8%) were gBRCA mutation carriers (*BRCA1*: *n* = 118, 14.7%; *BRCA2*: *n* = 9, 1.1%). The mutation prevalence was 32.9% in the age group 20–29 years compared to 6.9% in the age group 60–69 years. Logistic regression analysis revealed a significant increase of mutation frequency with decreasing age at diagnosis (odds ratio 1.87 per 10 year decrease, 95%CI 1.50–2.32, *p* < 0.001). gBRCA mutation risk was predicted to be > 10% for women diagnosed below approximately 50 years.

**Conclusions:**

Based on the general understanding that a heterozygous mutation probability of 10% or greater justifies gBRCA mutation screening, women with TNBC diagnosed before the age of 50 years and no familial history of breast and ovarian cancer should be tested for gBRCA mutations. In Germany, this would concern approximately 880 women with newly diagnosed TNBC per year, of whom approximately 150 are expected to be identified as carriers of a pathogenic gBRCA mutation.

## Background

Triple-negative breast cancer (TNBC) is characterized by lacking expression of oestrogen receptor (ER), progesterone receptor (PR), and human epidermal growth factor receptor type 2 (HER2). TNBC has been reported to account for 12–24% of all breast cancers and is associated with a hereditary disease cause [1–3]. While germline *BRCA1/2* (gBRCA) mutations are found in about 5% of all breast cancers, higher mutation rates are observed in TNBC patients depending on age of onset and the presence of a family history of breast and ovarian cancer [4]. Approximately 70% of breast cancers arising in *BRCA1* mutation carriers and up to 23% of breast cancers in *BRCA2* carriers are triple-negative [2]. Consequently, some guidelines suggest genetic gBRCA mutation testing in this particular group of patients. For example, the National Institute for Health and Care Excellence (NICE) guidelines recommend gBRCA testing if the combined *BRCA1* and *BRCA2* mutation carrier probability is expected to be at least 10% [5]. According to the National Comprehensive Cancer Network (NCCN) and the American Society of Breast Surgeons, gBRCA testing is generally advised for women with TNBC diagnosed at an age of ≤60 years, irrespective of a positive cancer family history.

It is less clear, however, up to which age at TNBC diagnosis women, who do not have a family history of breast and ovarian cancer, should be recommended genetic testing. This prompted us to determine the age-dependent prevalence of gBRCA mutations in women with unilateral TNBC and without any family history of breast or ovarian cancer.

## Methods

### Study population

The study population comprised a total of 802 women with TNBC, who reported not to have any relatives with breast or ovarian cancer in their families. Of these, 649 women were consecutively registered and documented between July 1999 and January 2016 in 15 specialized university centres of the German Consortium for Hereditary Breast and Ovarian Cancer (GC-HBOC). GC-HBOC collects data on families suspected of having hereditary breast and ovarian cancer based on a defined set of clinical ascertainment criteria [6]. At the time of data analysis, a total of 2029 women with TNBC were recorded in the GC-HBOC registry (ie., 1380 with and 649 without a family history of breast and ovarian cancer). Women with a TNBC diagnosis before age 36, who did not have any familial cancer history, were included since 1999, while women with a later TNBC diagnosis were collected mainly since 2011, resulting in some overrepresentation of women with younger age at TNBC diagnosis. Another 153 women were taken from the randomized controlled GeparSixto (GBG 66, clinicaltrials.gov identifier: NCT01426880) trial of the German Breast Group (GBG) [7, 8]. These women were consecutively recruited between August 2011 and December 2012. Women with syn- or metachronous bilateral breast cancer and/or ovarian cancer were excluded. Family history was collected and documented by a medical health professional (geneticist, genetic counselor, and/or gynecologist) after the diagnosis of TNBC. Pedigree information was required to encompass at least three generations.

The multicentre GC-HBOC registry and the multicentre GBG GeparSixto trial have been approved by the responsible ethics committees. Written informed consent to be enrolled in the GC-HBOC registry or the GBG GeparSixto trial, respectively, was obtained from all individuals whose data was used for the present analysis.

### Receptor status and mutation analysis

ER, PR and HER2 expression was determined according to the national pathology guidelines of the ‘AGO Breast Committee’, which adheres closely to international standards. Triple negativity was defined as immunohistochemical staining of less than 1% of nuclei for both ER and PR, and an immunhistochemical result (DAKO score) of 0 or 1+ for Her2/neu. Mutation analyses were performed using either next generation sequencing methods or denaturing high-performance liquid chromatography and high-resolution melting followed by direct Sanger-based sequencing of conspicuous fragments [9, 10]. If no deleterious sequence alterations were found in these analyses, samples were screened for large genomic alterations in the *BRCA1/2* genes by Multiplex Ligation-dependent Probe Amplification (MLPA) with the SALSA® MLPA® probemixes P002 for *BRCA1* and P045 for *BRCA2* (MRC-Holland, Amsterdam, The Netherlands) according to the manufacturer’s protocol. Mutations were classified according to the International Agency for Research on Cancer (IARC) system and considered pathogenic or likely pathogenic (class 4 or 5) based on literature evidence, multifactorial likelihood and functional analyses of the ENIGMA consortium that comprises genetic data of the GC-HBOC database [11, 12].

### Statistical analysis

Logistic regression was used to analyse the association between age of diagnosis and the presence of a gBRCA mutation. Ninety-five percent confidence intervals for frequencies were calculated applying Wilson’s score method. For the calculation of the expected annual number of gBRCA mutation carriers among women with TNBC and without any relatives with breast or ovarian cancer, data on the age-specific annual numbers of all breast cancers in Germany were obtained from pooled data of the German Epidemiological Cancer Registries (German Centre for Cancer Registry Data at the Robert Koch-Institute, Berlin, Germany). Age-specific frequencies of TNBC cases among all breast cancers were kindly provided by the Munich Cancer Registry (MCR, Munich, Germany). The age-specific proportions of TNBC without a positive family history among unselected TNBC were taken from Couch et al. [13]. *P*-values < 0.05 were considered significant. Statistical analyses were conducted using IBM SPSS Statistics for Windows Version 23.0 (IBM Corporation, Armonk, NY, USA) and R 3.3.2 for Windows (R Core Team, www.r-project.org).

## Results

A total of 802 patients were included in the study (649 from the GC-HBOC registry and 153 from the GBG randomized controlled GeparSixto trial). Basic patient characteristics are shown in Table 1. The median age at TNBC diagnosis was 40 (range 19–76) years and the overall prevalence of pathogenic gBRCA mutations was 15.8% (14.7% for *BRCA1* and 1.1% for *BRCA2*). *BRCA1* mutation carriers were younger at diagnosis (34 years) than *BRCA2* mutation carriers (47 years). Non-carriers had a median age at diagnosis of 42 years. Patients from the GeparSixto study were older at primary TNBC diagnosis compared to the patients from the GC-HBOC registry (48 vs. 38 years), and had a lower mutation prevalence (9.8% vs. 17.3%). The considerably lower age at TNBC diagnosis of GC-HBOC patients is explained by an overrepresentation of women diagnosed before the age of 36 years (see Materials). Table 2 depicts the age-group specific *BRCA1* and *BRCA2* mutation prevalence. The mutation prevalence increased with younger age at diagnosis. While in about one third of the very young patients (20 to 29 years) a gBRCA mutation was detected (33%), the prevalence was below 7% in older patients between 60 and 69 years.

**Table 1:** Basic patient characteristics

	Total *n* = 802	GC-HBOC *n* = 649	GBG *n* = 153
*BRCA* mutation status, no (%)			
negative	675 (84.2)	537 (82.7)	138 (90.2)
*BRCA1*	118 (14.7)	107 (16.5)	11 (7.2)
*BRCA2*	9 (1.1)	5 (0.8)	4 (2.6)
Age at diagnosis, years (median, range)	40.1 (19.5–76.2)	38.0 (19.5–76.2)	48.0 (21.0–74.0)
negative	42.0 (19.5–76.2)	38.7 (19.5–76.2)	48.5 (26.0–74.0)
*BRCA1*	34.1 (21.0–63.5)	33.5 (25.0–63.5)	41.0 (21.0–61.0)
*BRCA2*	47.2 (34.3–63.1)	47.1 (34.3–63.1)	54.0 (39.0–63.0)

**Table 2:** *BRCA 1/2* germline mutation prevalence by age group

	*n*	*BRCA1*		*BRCA2*		*BRCA1/2*	
		*n*	% (95%CI)	*n*	% (95%CI)	*n*	% (95%CI)
Age group (years)							
20–29	85	28	32.9 (23.9–43.5)	0	0.0 (0.0–4.3)	28	32.9 (23.9–43.5)
30–39	309	60	19.4 (15.4–24.2)	3	1.0 (0.3–2.8)	63	20.4 (16.3–25.2)
40–49	216	22	10.2 (6.8–14.9)	3	1.4 (0.5–4.0)	25	11.6 (8.0–16.5)
50–59	122	6	4.9 (2.3–10.3)	1	0.8 (0.1–4.5)	7	5.7 (2.8–11.4)
60–69	58	2	3.4 (1.0–11.7)	2	3.4 (1.0–11.7)	4	6.9 (2.7–16.4)
70–79	12	0	0.0 (0.0–24.2)	0	0.0 (0.0–24.2)	0	0.0 (0.0–24.2)
TOTAL	802	118	14.7 (12.4–17.3)	9	1.1 (0.6–2.1)	127	15.8 (13.5–18.5)

**Fig. 1:**
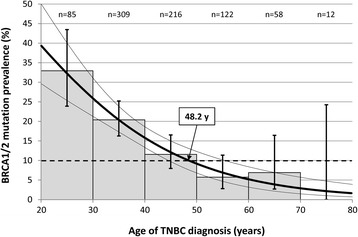
Association between age of TNBC diagnosis and gBRCA mutation prevalence. Grey shaded bars indicate the mutation prevalence in the according age group (error bars indicate 95% confidence intervals). The bold curve represents the mutation probability predicted by the logistic regression model (with 95% confidence band, thin curves). The dashed horizontal line depicts the 10% mutation probability, above which gBRCA mutation analysis is recommended

To assess the association between age of TNBC at diagnosis and gBRCA mutation prevalence, a logistic regression analysis was performed (Fig. 1). This analysis revealed a significant negative association between age at diagnosis and the presence of a gBRCA mutation (OR 1.87 per 10 year decrease, 95%CI 1.50 to 2.32, *p* < 0.001). At an age of approximately 48 years or younger, the predicted mutation probability was > 10%, which is the currently accepted international threshold to recommend gBRCA testing according to the NICE guidelines [5]. The origin of the data (GC-HBOC registry versus GBG GeparSixto trial) did not play a role as a significant confounder in the regression analysis (*p* = 0.823).

The performance of age at TNBC diagnosis to discriminate between gBRCA carriers and non-carriers, as characterized by the area under the curve of the receiver-operating-characteristic (ROC-AUC), was 67.0% (95%CI 62.1–71.9%). Recommendation of genetic testing only to TNBC patients diagnosed before the age of 50 years would result in a sensitivity of 96.9% (95%CI 92.2–98.8%) and a specificity of 9.8% (95%CI 7.8–12.3%).

Table 3 shows an estimation of the expected annual number of TNBC cases with a gBRCA mutation in the general German population, based on the age-specific absolute numbers of all breast cancers up to an age of 80 years and the expected rate of TNBC cases without family history. According to this calculation, the expected frequency of TNBC cases without family history among all breast cancers diagnosed below the age of 50 years would be 26.1% (882 among 3376 cases), with an expected number of 149 pathogenic gBRCA mutation carriers (positive predictive value of 16.9% among TNBC cases under 50 years). The expected number of gBRCA carriers among 2494 women with TNBC diagnosed at age 50 or above would amount to 109, corresponding to a negative predictive value of 95.6%.

**Table 3:** Expected annual number of women with TNBC and gBRCA mutation in Germany

Age group	Number of newly diagnosed BC cases in 2012 ^a^	% TNBC among all BC cases ^b^	% of TNBC w/o FH ^c^	Number of TNBC cases w/o FH	% *BRCA1/2* mutation carriers among TNBC cases w/o FH ^d^	Number of *BRCA1/2* mutation carriers among TNBC cases without FH
< 40	2462	19.8	62.2	302	26.3	79
40–49	9757	10.5	56.8	580	12.1	70
50–59	15,059	9.0	65.8	891	6.9	61
60–69	16,497	7.0	71.9	835	3.8	32
70–79	15,420	6.9	71.9	768	2.1	16

*FH* family history, *BC* breast cancer, *TNBC* triple-negative breast cancer

^a^German Centre for Cancer Registry Data at the Robert Koch-Institute, Berlin, Germany

^b^Munich Cancer Registry, MCR, Munich, Germany

^c^Couch et al. [13]

^d^present study

## Discussion

We conducted the present study to determine up to which age of TNBC diagnosis women without a family history of breast and ovarian cancer should undergo gBRCA testing if an expected mutation probability of at least 10% is used as decision threshold. This cut-off is currently used as general decision threshold to consider gBRCA testing in Germany.

Our analysis of 802 women revealed that this age cut-off lies at approximately 50 years. However, our analysis also showed that the discriminative performance of age at diagnosis as an exclusive predictor for the presence of a gBRCA mutation was comparatively low with an ROC-AUC of only 67%. If an age cut-off of 50 years would be chosen, the sensitivity would be 97%, with a specificity of 10%. If nationwide gBRCA mutation testing in Germany would be offered to women with newly diagnosed TNBC before the age of 50 years, we expect to identify around 150 additional mutation carriers among around 880 women to be tested per year, corresponding to a positive predictive value of approximately 17%. Among the larger number of approximately 2500 women diagnosed with TNBC at 50 years or later, a total of about 110 mutation carrying women would not be detected (4.4%; negative predictive value 96.6%), which is about 42% of all expected mutation carriers among TNBC cases up to the age of 80 years.

A number of earlier studies have investigated the gBRCA mutation prevalence in women with TNBC [13–22]. One study based on 207 TNBC cases unselected for family history has also employed logistic regression analysis to describe the association between age at diagnosis and mutation probability accounting for the presence or absence of family history [19]. In this study, the observed mutation prevalence was 6.3% (95%CI 1–12%), which is lower than found in our analysis. Couch et al. presented data on 1508 TNBC cases unselected for family history, for whom information on age at diagnosis and family history was complete [13]. In the subgroup of women with TNBC up to the age of 59 years without family history they reported a mutation prevalence of 11.2% (*BRCA1* 8.1% and *BRCA2* 3.0%).

Current guidelines regarding the age up to which TNBC patients should be offered gBRCA testing vary between countries. The NICE guideline recommends gBRCA mutation testing for women with TNBC and no family history diagnosed before 40 years of age, while the NCCN guideline recommends testing up to the age of 60 years. The normative definition of such decision thresholds depends on the expected mutation probabilities above which insurance carriers are willing to cover the costs for genetic testing. However, the definition of optimal BRCA genetic testing programs and decision cut-offs requires a thorough economic evaluation regarding their cost-effectiveness [23]. Such evaluations are currently not available for Germany but urgently needed.

A limitation of our study is the comparatively low sample size for women with an age at TNBC diagnosis of ≥60 years, resulting in large confidence intervals of the mutation prevalence estimates.

## Conclusions

Based on the understanding that a heterozygous mutation probability of ≥10% justifies gBRCA mutation screening, women with triple-negative breast cancer diagnosed before the age of 50 years and no familial history of breast and ovarian cancer should be tested for gBRCA mutations. In Germany, this would concern approximately 880 women with newly diagnosed TNBC per year, of whom approximately 150 are expected to be identified as pathogenic gBRCA mutation carriers.

## Abbreviations

BRCA1/2: Breast cancer gene 1 and 2
CI: Confidence interval
ER: Estrogen receptor
GBG: German Breast Group
gBRCA: germline BRCA1/2 mutation
GC-HBOC: German Consortium for Hereditary Breast and Ovarian Cancer
HER2: Human epidermal growth factor receptor 2
OR: Odds ratio
PR: Progesterone receptor
ROC-AUC: Area under the curve of the receiver operating-characteristic
TNBC: Triple negative breast cancer
